# Adenosquamous Carcinoma of Gallbladder with Unusual Prognosis: A Case Report

**DOI:** 10.31729/jnma.7923

**Published:** 2023-01-31

**Authors:** Siddinath Gyawali, Biraj Pokhrel, Deepak Sharma, Naveen Chandra Bhatta, Bishnu Prasad Kandel, Paleswan Joshi Lakhey

**Affiliations:** 1 Maharajgunj Medical Campus, Institute of Medicine, Kathmandu, Nepal; 2 Department of Surgical Gastroenterology, Tribhuvan University Teaching Hospital, Institute of Medicine, Kathmandu, Nepal

**Keywords:** *carcinoma*, *case reports*, *cholecystectomy*, *prognosis*

## Abstract

The adenosquamous carcinoma of the gallbladder is a rare variant accounting for only 1-4% of all primary gallbladder carcinoma. Regardless of the histological types, all gallbladder carcinomas have silent and rapid progression resulting in delayed diagnosis and poor prognosis. Even with medical and/or surgical interventions, the median survival of patients with adenosquamous carcinoma, one of the histological variants, is less than a year. However, we present a case of adenosquamous carcinoma with an unusually better prognosis. A 70-year-old female patient, after being diagnosed with gallbladder carcinoma was suggested for surgical resection but was lost to follow-up since then. Two years later, the patient presented and was managed with extended cholecystectomy. The slow progression and non-recurrence of the tumour during follow-up for two years after the surgery indicates a better prognosis in this case.

## INTRODUCTION

Gallbladder carcinoma (GBC) is uncommon cancer accounting for 1.2% of all global cancer and 1.7% of all cancer deaths.^1^ Its rapid and silent course results in late diagnosis and poor prognosis.^2^ The patient's survival for more than a year is highly unrealistic.^3,4^ Among the histological variants, adenosquamous carcinoma (ASC-GB) is associated with early metastasis and poorer prognosis.^5^ Herein, we report a case of a patient who was diagnosed with GBC and was lost to follow-up. Two years later, she was found to have a resectable gallbladder mass and was managed with extended cholecystectomy. Biopsy confirmed an ASC-GB.

## CASE REPORT

A 70-year-old female presented to Tribhuvan University Teaching Hospital (TUTH) with complaints of on and off abdominal pain for three months, insidious in onset, and increasing severity and frequency. The pain which was initially localized to the right upper quadrant of the abdomen gradually became generalized. There was no history of nausea, vomiting, fever, shortness of breath, loss of appetite, weight loss or jaundice.

**Figure 1:**
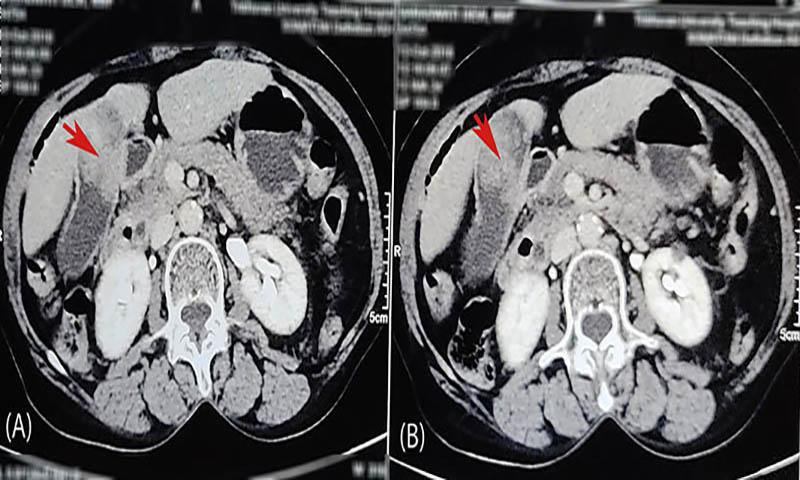
A, B) Contrast-enhanced CT scan of abdomen-pelvis done two years back showing enhancing mass in the neck of the gallbladder (shown by the red arrows).

The patient had presented to us two years before with similar complaints of abdominal pain in the right upper quadrant for a day and an episode of vomiting. She had no fever, and her bowel and bladder habits were normal. Her examination at that time revealed no icterus, and the abdomen was soft and non-tender without any palpable mass. The ultrasonography of her abdomen showed an approximately 4.2x2.7 cm^2^ sized hyperechoic lesion in the lumen of the gallbladder arising from its posterior wall suggestive of gallbladder mass. Contrast-enhanced computed tomography (CECT) scan of her abdomen was then carried out, which indicated features suggesting carcinoma of the gallbladder involving its body and fundus along with segment IV B of the liver (Figure 1).

She was suggested to carry out computed tomography (CT) scan of her chest and tests for tumour markers (carcinoembryonic antigen (CEA), carbohydrate antigen (CA) 19-9). She was planned for an extended cholecystectomy but she was lost to follow-up since then.

The examination of the patient in the second visit revealed fair general condition, vital signs within normal limits, and tenderness over her right hypochondrium. She had leukocytosis with 70% neutrophils, anaemia, deranged liver function and normal renal function (Table 1).

**Table 1:** Laboratory findings at the time of admission.

Laboratory tests	Results
**Hematology**	
Hemoglobin	9.2 gm%
Packed cell volume	27.5%
RBCs	3.0 million/cubic mm
Leucocytes	13,600
Platelets	433,000
**Liver function test**	
Total bilirubin	28 μMol/L
Direct bilirubin	10 μMol/L
Alanine aminotransferase	15 U/L
Aspartate aminotransferase	21 U/L
Alkaline phosphatase	276 U/L

**Figure 2:**
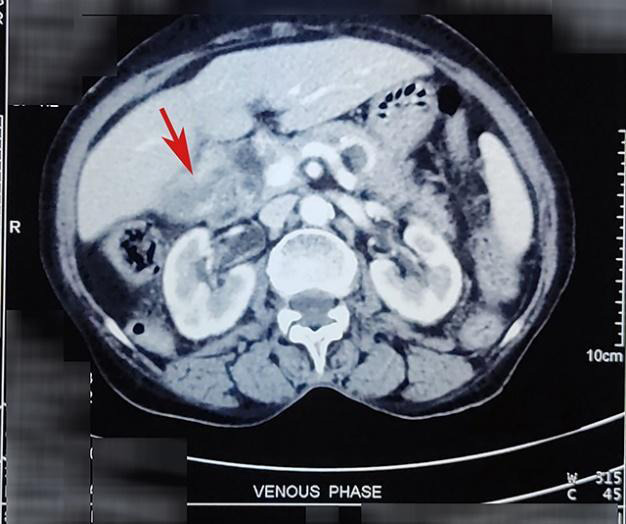
CECT abdomen-pelvis prior to the surgery showing non-progression of the mass in the neck of the gallbladder (shown by red arrow).

CECT of the abdomen and pelvis was carried out again, which showed an ill-defined heterogeneously enhancing mass (6x3.2x2.7 cm^3^) in the gallbladder with asymmetrical thickening of its body and neck (maximum 18.3 mm) (Figure 2).

She was planned for staging laparoscopy and extended cholecystectomy. As the patient had COPD, consultation was made with the respiratory and cardiology department for optimization before the surgery.

**Figure 3:**
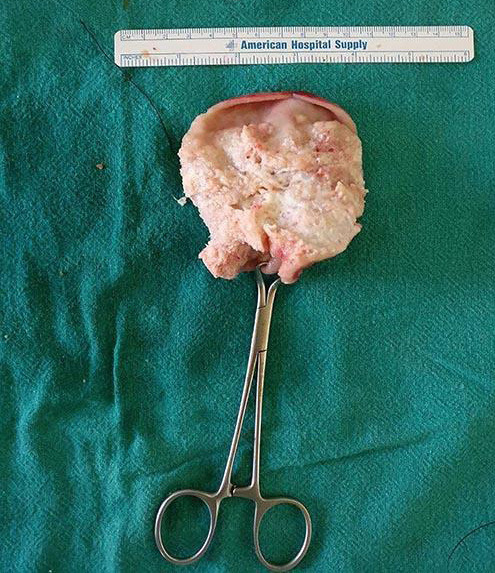
Gross specimen of the gallbladder showing papillary growth pattern in the neck and body.

Laparotomy was done with a right subcostal incision. There was a mass arising from the fundus of the gallbladder. There were no ascites, and no liver or peritoneal deposits. Extended cholecystectomy was done, and the resected specimen containing the gallbladder, along with the liver margin, cystic duct margin and periportal tissue was sent for histopathological examination (Figure 3).

**Figure 4:**
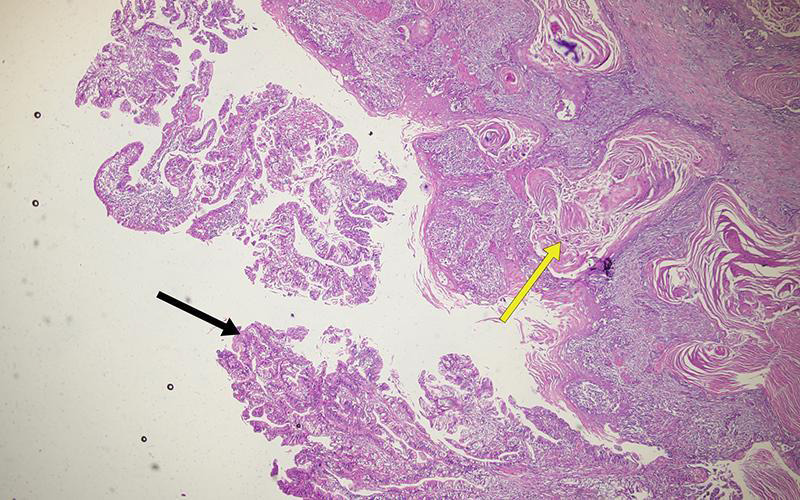
Magnified view (hematoxylin and eosin stain, x100) of the specimen showing adenomatous (black arrow) and squamous components (yellow arrow).

The histopathology of the specimen confirmed adenosquamous carcinoma of the gallbladder located along its body and fundus, with the squamous component being more than 90% (Figure 4).

The maximum dimension of the tumour was identified to be 8 cm. The tumour had invaded the perimuscular connective tissue along the hepatic side; the cystic duct and the liver margins were free of the tumour. In addition, perineural and lymphovascular invasions by the tumour were identified. Out of twelve lymph nodes dissected, one was positive for metastasis. The tumour was staged as pT2bN1. During the follow-up for two years, she was asymptomatic, and the ultrasound of the abdomen did not show any features of tumour recurrence.

## DISCUSSION

The histological types of gall bladder and extrahepatic bile duct malignancies are adenocarcinoma (AC; 9095%), squamous cell carcinoma (SCC), adenosquamous carcinoma (ASC; 1-5%), neuroendocrine carcinoma, mixed neuroendocrine non-neuroendocrine neoplasm and undifferentiated carcinoma.^2,6,7^ As with any variants of GBC, patients with ASC-GB may present with abdominal pain, weight loss, loss of appetite, nausea and vomiting, lumpy sensation in the right upper quadrant, and jaundice. The ultrasonography of the abdomen shows features suggesting a gallbladder mass which is further strengthened by the CT scan which also determines the exact location, extent, and local or distant spread of the tumour.^2^ Complete surgical resection is the treatment of choice in all cases of GBC; however, most patients present at an advanced stage when the tumour is no longer resectable.^4^ The surgical treatment options for GBC include extended cholecystectomy with the removal of 2 cm of normal liver around the gallbladder along with lymph node dissection along the hepatoduodenal ligaments. The CECT scan in our case showed a mass limited to the gallbladder, so the resection of the gallbladder along with 2 cm of liver bed and standard lymphadenectomy was carried out.

The prognosis of GBC is determined by the stage of the disease and its histological type. In a study among 69 cases with ASC-GB, the overall 5-year survival rate was estimated to be less than 5% with a median survival of 8.5 months.^5^ On the contrary, a comparative study between AC-GB and SCC-GB with ASC-GB depicted non-significant differences in prognosis among those histological variants with a median survival of less than 7 months.^8^ Though incongruities exist among the studies regarding the relationship between the histological type and prognosis of malignancy, these studies agree that all GBCs have poor prognoses with a median survival of less than a year.^2,4,9^

However, contrary to these reported statistics, our patient was lost to follow-up after been diagnosed with GBC which was found to be non-progressive. Even after 2 years, the tumour was still resectable, and there were no signs and symptoms of tumour recurrence in the 2-year follow-up after the surgery suggesting that the tumour had a good prognosis in our case. The invasion of only perimuscular tissue along the hepatic side by the tumour but not the cystic margin and the liver, and the good biology of the tumour might have aided in the better prognosis.

## References

[1] Rawla P, Sunkara T, Thandra KC, Barsouk A (2019). Epidemiology of gallbladder cancer.. Clin Exp Hepatol..

[2] Misra S, Chaturvedi A, Misra NC, Sharma ID (2003). Carcinoma of the gallbladder.. Lancet Oncol..

[3] Gulwani HV, Gupta S, Kaur S (2017). Squamous cell and adenosquamous carcinoma of gall bladder: a clinicopathological study of 8 cases isolated in 94 cancers.. Indian J Surg Oncol..

[4] Song HW, Chen C, Shen HX, Ma L, Zhao YL, Zhang GJ (2015). Squamous/adenosquamous carcinoma of the gallbladder: analysis of 34 cases and comparison of clinicopathologic features and surgical outcomes with adenocarcinoma.. J Surg Oncol..

[5] Davis T, Moudy P, Abdelgawad M, Barghuthi L, Ismael H (2021). Gallbladder adenosquamous carcinoma: a case report and literature review.. J Surg Case Rep.

[6] Henson DE, Albores-Saavedra J, Corle D (1992). Carcinoma of the gallbladder.. Histologic types, stage of disease, grade, and survival rates. Cancer..

[7] Nagtegaal ID, Odze RD, Klimstra D, Paradis V, Rugge M, Schirmacher P (2020). The 2019 WHO classification of tumours of the digestive system.. Histopathology..

[8] Chan KM, Yu MC, Lee WC, Jan YY, Chen MF (2007). Adenosquamous/squamous cell carcinoma of the gallbladder.. J Surg Oncol..

[9] Kondo M, Dono K, Sakon M, Shimizu J, Nagano H, Nakamori S (2002). Adenosquamous carcinoma of the gallbladder.. Hepatogastroenterology..

